# Development of Immune-Regulatory Pseudo-Protein-Coated Iron Oxide Nanoparticles for Enhanced Treatment of Triple-Negative Breast Tumor

**DOI:** 10.3390/nano15131006

**Published:** 2025-06-30

**Authors:** Ying Ji, Juan Li, Li Ma, Zhijie Wang, Bochu Du, Hiu Yee Kwan, Zhaoxiang Bian, Chih-Chang Chu

**Affiliations:** 1 Research Institute for Intelligent Wearable Systems, Hong Kong Polytechnic University, Hong Kong SAR, China; li-lili.ma@connect.polyu.hk (L.M.);; 2 CAS Key Laboratory for Biomedical Effects of Nanomaterial & Nanosafety, Institute of High Energy Physics, Chinese Academy of Sciences, Beijing 100049, China; 3 College of Biomass Science and Engineering, Sichuan University, Chengdu 610065, China; 4 School of Chinese Medicine, Hong Kong Baptist University, Hong Kong SAR, China; hykwan@hkbu.edu.hk (H.Y.K.); bzxiang@hkbu.edu.hk (Z.B.); 5 Biomedical Engineering Field, Cornell University, Ithaca, NY 14853, USA; cc62@cornell.edu

**Keywords:** poly (ester urea urethane), iron oxide nanoparticles, arginine, immune regulation, macrophage re-polarization

## Abstract

Triple-negative breast cancer (TNBC) frequently evades immune recognition and elimination, resulting in an immunosuppressive microenvironment. The phagocytic activity of tumor-associated macrophages underscores the development of nanomaterials as a promising strategy to target these macrophages and modulate their polarization, thereby advancing immunotherapy against TNBC. This research developed functional polymers that are complexed with therapeutic molecules as a coating strategy for iron oxide nanoparticles. An arginine-based poly (ester urea urethane) polymer complexed with a macrophage-polarizing molecule (APU-R848) could provide a synergistic effect with iron oxide nanoparticles (IONPs) to stimulate the M1-polarization of macrophages at the tumor site, resulting in a versatile nano-platform for immune regulation of TNBC. In the 4T1 in vivo breast tumor model, the APU-R848-IONPs demonstrated an improved intratumoral biodistribution compared to IONPs without a polymer coating. APU-R848-IONPs significantly reversed the immune-suppressive tumor environment by reducing the M2/M1 macrophage phenotype ratio by 51%, associated with an elevated population of cytotoxic T cells and a significantly enhanced production of tumoricidal cytokines. The activated immune response induced by APU-R848-IONP resulted in a significant anti-tumor effect, demonstrating an efficacy that was more than 3.2-fold more efficient compared to the controls. These immune-regulatory pseudo-protein-coated iron oxide nanoparticles represent an effective nano-strategy for macrophages’ regulation and the activation of anti-tumor immunity, providing a new treatment modality for triple-negative breast cancer.

## 1. Introduction

The challenges faced by patients with triple-negative breast cancer (TNBC) include the absence of targeted therapies and a poor prognosis, both of which have fostered a major effort to discover new therapeutic and diagnostic approaches [1,2]. As an emerging anti-tumor therapeutic strategy, immunotherapy leverages the host immune response instead of toxic chemotherapeutic agents for tumors’ elimination. This approach could provide a new perspective for TNBC’s treatment [3,4].

Macrophages serve as a primary cell population that interacts with foreign substances. In addition to their phagocytic activity, macrophages play an essential role in both innate and adaptive immunity. The modulation of macrophages represents a promising and innovative strategy for anti-tumor treatment, enhancing immune surveillance in the tumor microenvironment to eliminate tumor cells [5]. In tumors’ progression, macrophages and tumor cells engage in a complex interplay. Macrophages produce chemokines that inhibit cancer cells; however, in most types of solid tumors, they can also promote angiogenesis, contributing to tumors’ progression. By regulating the inducible nitric oxide synthase (iNOS) and arginase pathways of arginine metabolism, macrophages can perform either destructive or constructive functions in pathological processes [6,7]. Due to the phagocytic activity of macrophages, foreign nanomaterials engage in a first-line interaction with immune cells as soon as they enter the physiological system [8,9]. Therefore, engineering nanomaterials presents a promising strategy for targeting macrophages and influencing macrophages’ polarization. The integration of nanotechnology with immunology has significantly impacted cancer immunotherapy, enabling the precise modulation of immune cells and tumor microenvironments [10]. Recent advances in molecularly targeted nanotherapies further highlight the potential of engineered nanomaterials to enhance immune activation while minimizing off-target effects [11]. Multifunctional nanoparticles can simultaneously deliver immunomodulators and target specific cellular pathways, offering synergistic anti-tumor responses [12].

Amino acid-based poly (ester urea urethane) is composed of amino acid-based ester urea blocks and urethane blocks. The hydrolyzable ester linkage in poly (ester urea urethane) could enhance the degradation rate and improve the payload release profile. The urethane moieties can introduce additional pendant groups for functionalization. Amino acid-based poly (ester urea urethane) comprises a broad range of functional components and could provide a versatile polymeric nano-platform [13]. Previous studies conducted by our research group have developed an amphiphilic polyurethane copolymer based on arginine (Arg) and leucine (Leu) for the delivery of doxorubicin in HeLa cells [14]. An arginine-based cationic poly (ester urea urethane) modified with poly (ethylene glycol)-folate (PEG-folate) was developed for the in vitro delivery of gambogic acid in HeLa and A549 cells [15]. Among versatile pseudo-proteins, arginine-based poly (ester amide) and poly (ester urea urethane) can modulate arginine metabolism in macrophages through both the iNOS and arginase pathways [16,17]. As a crucial building block and signaling molecule, arginine plays a regulatory role in multiple physiological processes. Arginine can impact the activation states of macrophages (M1 versus M2), thereby directing their immune responses [7]. Macrophage activation is closely associated with various pathological processes [18], including immune surveillance and tumor inhibition [19]. Arginine-based pseudo-protein could regulate the homeostasis between M1 and M2 macrophages. M1 macrophages secrete inflammatory cytokines, such as TNF-α, which promote Th1 responses that enhance resistance against tumor growth. Arginine-based pseudo-proteins also stimulate the production of nitric oxide (NO), resulting in cell cycle arrest, tumor cell cytostasis, or apoptosis and further sensitizing cancer cells to TNF-α-induced cytotoxicity [20,21]. Therefore, arginine-containing biodegradable pseudo-proteins can promote the inflammatory activation of macrophages, augmenting their inflammatory response toward cancer cells and hence providing a synergistic anti-tumor effect with other therapeutic reagents.

Iron oxide nanoparticles have been extensively studied for their applications in cancer diagnosis and therapy. Due to the aggregation potential of bare iron oxide nanoparticles, a polymeric coating strategy has been developed to enhance the colloidal stability of iron oxide nanoparticles in physiological environments [22,23]. In addition to applications as a contrast agent for magnetic resonance imaging (MRI), the therapeutic potential of iron oxide nanoparticles, especially their immune-regulatory interaction with the microenvironment of tumors, provides new application potential [24]. Researchers have investigated the potential effects of iron oxide nanoparticles on the polarization of macrophages into pro-inflammatory M1 phenotypes [25,26].

Iron oxide nanoparticles could induce the production of reactive oxygen species (ROS) and modulate intracellular redox and iron metabolism to impact macrophages [27]. However, uncoated iron oxide nanoparticles may exhibit cytotoxic effects by inducing oxidative stress, lipid peroxidation and ROS overproduction, which can inadvertently exacerbate inflammation or cause off-target toxicity [28]. Studies have highlighted the importance of the surface coating of iron oxide nanoparticles, which could have an essential impact on the immune-regulatory outcome in cancer immunotherapy [29]. Recent studies also demonstrate that surface-engineered nanoparticles alone can directly modulate macrophage polarization through ROS-dependent pathways, independent of therapeutic cargo [30]. Concurrently, R848 self-assembled nanoparticles have emerged as potent immunomodulators, significantly enhancing anti-tumor immunity by synergistically activating TLR7/8 pathways and re-polarizing tumor-associated macrophages [31].

This research aims to integrate immune-regulatory biomaterial technology with iron oxide nanoparticles to develop a new immunotherapeutic strategy for TNBC. The research focused on the structural design of arginine-based poly (ester urea urethane) (abbreviated as APU) and the formulation of therapeutic molecules, to develop a multifunctional coating strategy for iron oxide nanoparticles. Pseudo-protein-coated iron oxide nanoparticles have the potential to modulate macrophage polarization towards the M1 phenotype in the tumor microenvironment. This study aims to investigate the interactions between nanoparticles and the immune system to improve cancer immune therapy for TNBC.

## 2. Materials and Methods

### 2.1. Chemicals and Reagents

L-Arginine hydrochloride, ethylene glycol, 1,6-hexanediol, p-toluene sulfonic acid (TsOH) monohydrate, glycerol α-monoallyl ether (GAE), hexamethylene diisocyanate (HDI), 2,6 di-tert-butyl-4-methyl phenol (BHT), stannous 2-ethyl-hexanoate, 2,2′-Azobis-isobutyronitrile (AIBN), 4-mercaptobenzoic acid (4-MBA), R848 (Resiquimod) and lipase from porcine pancreas (100–650 U/mg) were purchased from Sigma Aldrich, St. Louis, MO, USA. Dimercaptosuccinic acid (DMSA)-functionalized Fe_3_O_4_ iron oxide nanoparticles (DMSA-IONP) were purchased from Nanjing Muke Nanotechnology Co., Ltd. (Nanjing, China). Toluene, dimethyl sulfoxide (DMSO), dimethylformamide (DMF), isopropyl alcohol, ethyl acetate and other HPLC-grade solvents were from JT Baker, Phillipsburg, NJ, USA. Rhodamine-labeled derivative of APU polymer was prepared based on previously published research [14].

### 2.2. Synthesis

The detailed synthesis procedures for arginine-based poly (ester urea urethane) (APU), 4-MBA-functionalized APU (4-MBA-APU) and the resulting R848-complexed APU (APU-R848) polymers are included in the Supplementary Materials. The as-synthesized polymers were dissolved in DMSO-*d6* (Cambridge Isotope Laboratories), and the ^1^H-NMR spectra were collected on a Bruker Advance-III 400 MHz spectrometer (Bruker, Ettlingen, Germany). The molecular weight of the polymers was characterized by gel permeation chromatography (GPC) on a Water Breeze 1525 system (Waters Corporation, Milford, MA, USA) using DMF as eluent.

### 2.3. Coating of Iron Oxide Nanoparticles with APU-R848

To prepared APU-R848-coated IONPs (APU-R848-IONPs), APU-R848 was dissolved in DMSO (0.5 mg/mL, 1 mL). Subsequently, 0.5 mL of DMSA-IONP (1 mg Fe/mL in deionized water) was added dropwise to the above solution and stirred for 6 h. The solution was dialyzed against deionized water for 48 h. The obtained nanoparticle solution was concentrated with Amicon^®^ Ultra-15 centrifugal filter unit (MWCO = 100 kDa, Merck Millipore, Burlington, MA, USA) and stored for further use. The morphology of the nanoparticles was characterized on an FEI Tecnai T12 transmission electron microscope (FEI, Hillsboro, OR, USA) at an operating voltage of 120 kV. The size and zeta potential were tested on Zeta Sizer Nano ZS (Malvern, UK).

To characterize the degradation of the polymer coating and the release of R848, APU-R848-IONPs were dispersed in a phosphate buffer (pH 7.4) containing 3 mg/mL of lipase. The solution was dialyzed (MWCO = 100 kDa) against a phosphate buffer containing 0.5% Tween 80 at 37 °C under shaking (100 rpm). At predetermined time intervals, 1 mL of the solution outside the dialysis tubing was collected and replaced with fresh media. The concentration of released R848 was determined by high-performance liquid chromatography [32] (Agilent, Santa Clara, CA, USA) with a UV detector (254 nm) and a C8 column. The mobile phase consisted of the mixture of water (0.1% *v*/*v* trifluoroacetic acid) and acetonitrile (0.1% *v*/*v* trifluoroacetic acid) with a gradient elution (20–95%) at a flow rate of 1.0 mL/min.

### 2.4. In Vitro Study

Cell culture. RAW 264.7 murine macrophages were cultured in complete DMEM containing 10% fetal bovine serum (FBS) and 1% penicillin-streptomycin at 37 °C with 5% CO_2_. Bone marrow-derived macrophages (BMDMs) were collected from C57BL/6 mice (female, 8 weeks old). Bone marrow cells were flushed from the femurs and tibias and treated with diluted RBC lysis buffer. The bone marrow cells were cultured in complete DMEM supplemented with 10% FBS, 100 U/mL penicillin, 100 μg/mL streptomycin, 2 mM L-glutamine, 5 mM HEPES (pH 7.4) and 15% L929-conditioned medium. 4T1 breast cancer cells (CRL-2539) were cultured in RPMI-1640 supplemented with 10% FBS and 1% penicillin-streptomycin.

Viability study. RAW264.7 macrophages were incubated with APU-R848-IONPs at various Fe concentrations ranging from 25 to 300 μg Fe/mL. The viability of the cells was assessed using the CellTiter MTS assay kit (Promega, Madison, WI, USA).

Endocytosis detection. BMDMs were incubated with rhodamine-labeled APU-R848 or rhodamine-labelled APU-R848-IONPs for 4 h. Following incubation, BMDMs were stained with Lysotracker Green DND-26 (ThermoFisher, Waltham, MA, USA), and the intracellular fluorescence was visualized by confocal microscope (Leica TCS SP8, Leica, Wetzlar, Germany). BMDMs incubated with APU-R848 alone, IONPs alone or APU-R848-IONP were harvested, washed with PBS and counted. The cells were digested in 70% nitric acid: 36% hydrochloric acid (1:1 *v*/*v*) at 70 °C for 1 h. The Fe content per cell was measured by inductively coupled plasma optical emission spectroscopy (ICP-OES, Agilent 700, Agilent, Santa Clara, CA, USA).

Co-culture of 4T1 cells and BMDMs. BMDMs were seeded in the upper compartment Transwell insert of a 24-well Transwell culture system (0.4 μm pore size) and treated with APU-R848-IONP for 24 h. The macrophage-seeded Transwell inserts were then transferred into a new 24-well plate seeded with 4T1 cells. This co-culture system was maintained using RPMI-1640 supplemented with 10% FBS and 1% penicillin-streptomycin. After 48 h of co-culturing, the viability of the 4T1 cells in the bottom compartment was determined by MTS assay.

### 2.5. Macrophage Polarization

BMDMs were first differentiated into M2 macrophages by 10 ng/mL IL-4 for 24 h, followed by culturing with phosphate-buffered saline, APU-R848 alone, IONPs alone or APU-R848-IONPs for 24 h. The equivalent Fe content of APU-R848-IONPs in cell culture media was 60 μg Fe/mL. After washing and centrifugation, the cells were resuspended in PBS containing 0.2% bovine serum albumin (BSA) and stained with anti-CD206 (FITC-conjugated, MA5-16870, ThermoFisher) and anti-CD80 (PE-conjugated, MA5-17935, ThermoFisher) for 30 min at 4 °C. The cells were washed, resuspended in FACS buffer and analyzed on a flow cytometer (BD FACS CANTO II, BD Biosciences, San Jose, CA, USA). The total RNA was extracted using the RNeasy Kit (Qiagen, Hilden, Germany), and the mRNA expression levels of iNOS and Arg-1 were analyzed through quantitative real-time PCR (RT-PCR) with iTaq Universal SYBR Green Supermix (Bio-Rad, Hercules, CA, USA). iNOS forward primer: 5′-GTTCTCAGCCCAACAATACAAGA-3′; iNOS reverse primer: 5′-GTGGACGGGTCGATGTCAC-3′; Arg-1 forward primer: 5′-CTCCAAGCCAAAGTCCTTAGAG-3′; Arg-1 reverse primer: 5′-AGGAGCTGTCATTAGGGACATC-3′ [33].

### 2.6. In Vivo Animal Model

All animal experiments were approved by the Animal Subjects Ethics Sub-committee of Hong Kong Polytechnic University (No. 20–21/170-ITC-R-NSFC). The 4T1 TNBC model was established by inoculating 0.5 × 10^7^ 4T1 cells (50 μL, 50% *v*/*v* Matrigel in PBS) into the second right mammary fat pad of female Balb/c mice (8 weeks). Tumor growth was monitored using a digital caliper. Tumor size was monitored by measuring the length and width with an electronic caliper and was calculated according to the formula: tumor size = (length × width^2^)/2.

### 2.7. Biodistribution Study

Biodistribution of Fe. Tumor-bearing mice received an intravenous injection of either IONPs alone or APU-R848-IONPs (at an equivalent Fe dose of 10 mg/kg). At 4 h or 24 h post injection, tumors and the major organs (heart, liver, spleen, lung, kidneys) were collected. The collected tissue samples were weighed and digested overnight with 70% nitric acid: 36% hydrochloric acid (1:1 *v*/*v*) at 70 °C. The solution was filtered, and the Fe content was measured by ICP-OES. The Fe content in each organ was normalized to the total injected Fe content to characterize the biodistribution (n = 3).

### 2.8. Anti-Tumor Efficacy

Intravenous injections of various treatments, including saline, APU-R848 alone, IONPs alone or APU-R848-IONPs (n = 4), were administered when the tumor size reached 100 mm^3^. The Fe dose was 5 mg/kg/injection, and the corresponding R848 dose was 1.1 mg/kg/injection. A total of three injections were administered on days 1, 3 and 5. Tumor size was monitored. Mice were euthanized on day 21. The major organs and tumors were collected, fixed in 10% formalin, embedded in paraffin and sectioned at 5 µm for hematoxylin and eosin (H&E) staining. For the survival test, 4T1 tumor-bearing mice that received various treatments were monitored for 35 days. During the survival test period, when the tumor burden exceeded the maximum size permitted by animal welfare regulations [34], the mice were euthanized, and the survival rates were recorded.

### 2.9. Immunohistochemistry

Tumor sections were incubated with anti-cleaved caspase-3 (1:400, cat#9664), anti-CD31(1:200, #77699), anti-F4/80 (1:200, cat#70076), anti-CD206 (1:200, cat#24595), anti-CD86 (1:100, #91882) and anti-CD8α (1:400, #98941), followed by incubation with HRP-conjugated secondary antibodies and 3,3′-diaminobenzidine, and counterstained with hematoxylin. The antibodies were supplied by Cell Signalling Technology. The slides were scanned using a Digital Pathology Scanner (Leica BOND RX, Leica, Wetzlar, Germany). The expression of antigens was quantified by dividing the number of positive events by the total number of events, using ImageScope software (version 12.4.6, Aperio, Reston, VA, USA).

### 2.10. ELISA Detection

The collected tumor tissues were weighed and homogenized. Protein content was extracted in Tissue Extraction Reagent I (ThermoFisher) supplemented with 1 mM PMSF and Pierce protease inhibitor cocktail (ThermoFisher). Tissue lysates were prepared through centrifugation, and the protein concentration was measured by a Pierce BCA Protein Assay Kit (ThermoFisher). Cytokines in the tumor lysates were measured using a mouse IL-12 p70 Quantikine ELISA Kit (M1270, R&D Systems, Minneapolis, MN, USA), mouse TNF-α Quantikine ELISA Kit (MTA00B) and mouse IL-6 Quantikine ELISA kit (M6000B, R&D Systems).

### 2.11. Statistical Analysis

The data are presented as mean values with standard deviations. All data were analyzed using one-way ANOVA, followed by Tukey’s multiple comparison tests, and *p* < 0.05 was used for statistical significance.

## 3. Results and Discussion

### 3.1. Development of Immune-Regulatory Pseudo-Protein-Coated Iron Oxide Nanoparticles

R848 is demonstrated as a potent macrophage-regulatory molecule that can drive the M1-polarization of macrophages and alter the tumor immune microenvironment toward an M1 phenotype [35]. The reprogrammed macrophages, including tumor-associated macrophages (TAMs), play a crucial role in activating anti-tumor immunity. R848 promotes the T-helper 1 (Th1) immune response and enhances the production of multiple cytokines, particularly pro-inflammatory cytokines that could have anti-tumor effects. The immunostimulatory activity of R848 can stimulate the host immune surveillance to eliminate tumor cells. However, the limited water solubility of R848 significantly restricts its bioavailability in biomedical applications.

**Figure 1:**
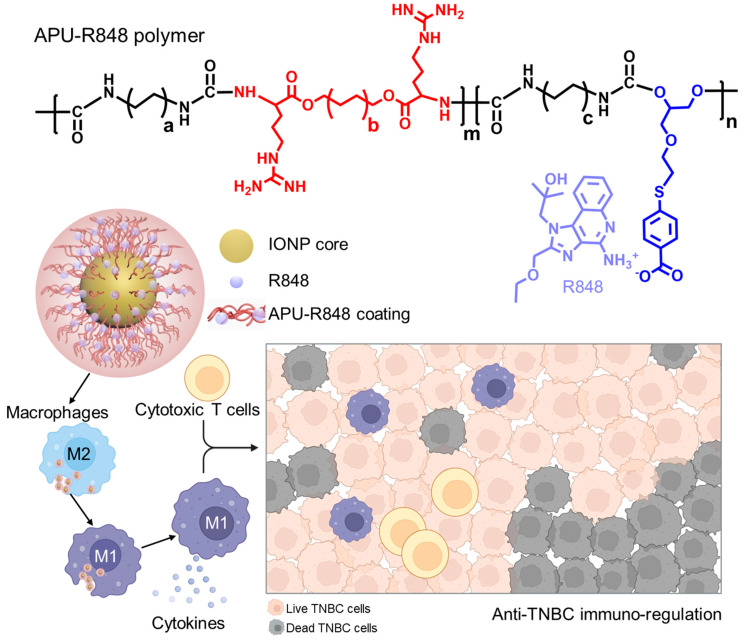
Schematic illustration of immune-regulatory pseudo-protein-coated iron oxide nanoparticles. The arginine-based poly (ester urea urethane) (APU) polymer was complexed with R848 through electrostatic and π–π interactions. Black segments: Non-arginine urea urethane segments in APU polymer backbone. Red segments: Arginine-containing ester segments in APU polymer backbone. Blue segments: 4-MBA-containing segments to complex with R848. The APU-R848 polymers formed a stable coating onto the IONP core, resulting in a macrophage-regulatory APU-R848-IONP to promote the M1-polarization of macrophages and stimulate anti-tumor immunity in TNBC.

Several nano-delivery vehicles have been developed for the delivery of R848, including liposomes [36], polyamidoamine dendrimers [33], T cell-targeting PEG-PLGA nanoparticles [37], tocopherol-modified hyaluronic acid [33] and platelet-cloaked nanoparticles [38]. Studies have explored the therapeutic potential of R848 in enhancing anti-tumor immunity. With the purpose of exploring the combinational potential of R848 with other immune-regulatory moieties, this study developed an arginine-based poly (ester urea urethane) coating polymer to improve the bioavailability of R848 and incorporate R848 into an iron oxide nanoparticle system. The general chemical structure of the arginine-based poly (ester urea urethane) (abbreviated as APU) is shown in Figure 1 and Figure S1 (Supplementary Materials). The double bond in APU polymers can be functionalized to form a complex with R848 through multiple interactions, including electrostatic interaction, π–π interaction and physical encapsulation. In addition to the complexation of R848, the urethane moieties in APU provided a good coating performance on metal oxide nanoparticless. Therefore, this study explored a coating of APU-R848 to develop functionalized iron oxide nanoparticles.

### 3.2. Synthesis and Functionalization of APU-R848 Polymer

The synthesis route for the APU-R848 polymer is included in Figure S2 (Supplementary Materials). The modification of APU was performed via thiol-ene click chemistry, which involved the reaction between the allyl groups in APU and the thiol groups in 4-mercaptobenzoic acid (4-MBA). The resulting 4-MBA-APU was an intermediate polymer in which the weakly basic amine groups could form an electrostatic interaction with the carboxylates in 4-MBA. Furthermore, the fused cyclic hydrocarbons in R848 could form a π–π interaction with 4-MBA to form an R848-complexed APU polymer (APU-R848).

The chemical structure of 4-MBA-APU was characterized by ^1^H-NMR in *d6*-DMSO as shown in Figure S3 (Supplementary Materials). After modification, the APU exhibited a loss of vinyl peaks in the chemical shift range of 5 to 6 ppm, indicating the consumption of double bonds through the thiol-ene reaction with 4-MBA. New peaks at 7 to 8 ppm, attributed to phenyl groups from 4-MBA, were observed in 4-MBA-APU, which demonstrated a chemical conjugation. The molecular weights of the 4-MBA-APU and APU-R848 polymers were characterized by gel permeation chromatography (GPC) with DMF as the eluent (Figure S4, Supplementary Materials). The weight average molecular weight of 4-MBA-APU was detected as 22,350 Da (Đ = 3.22). The weight average molecular weight of APU-R848 was detected as 26,765 Da (Đ = 2.59).

### 3.3. Coating of APU-R848 Polymers onto Iron Oxide Nanoparticles

The as-developed APU-R848 was then explored as a coating strategy for DMSA-IONP. The procedures of coating APU-R848 polymers onto IONPs were optimized by tuning various structural features. As summarized in Figure S5 (Supplementary Materials), factors including the ratio between arginine- and R848-containing blocks, -(CH_2_)- type, polymer to IONP ratio and concentrations, etc., were optimized based on the efficiency of the coating, stability of the coated IONP and proper content of arginine and R848. The optimized APU-R848 structure comprised a molar ratio of 1:1 between arginine and R848-containing blocks, each featuring -(CH_2_)_6_- hydrocarbon segments situated between urethane and ester groups along the polymer chains. In addition to polymer structure, the coating procedures involving the optimized APU-R848 to IONP feed ratio (1:1 *w*/*w*), as well as an IONP concentration of 0.5 to 1 mg Fe/mL, etc., (Figure S5, Supplementary Materials), adopted for the preparation of APU-R848-IONP in the following studies.

**Figure 2:**
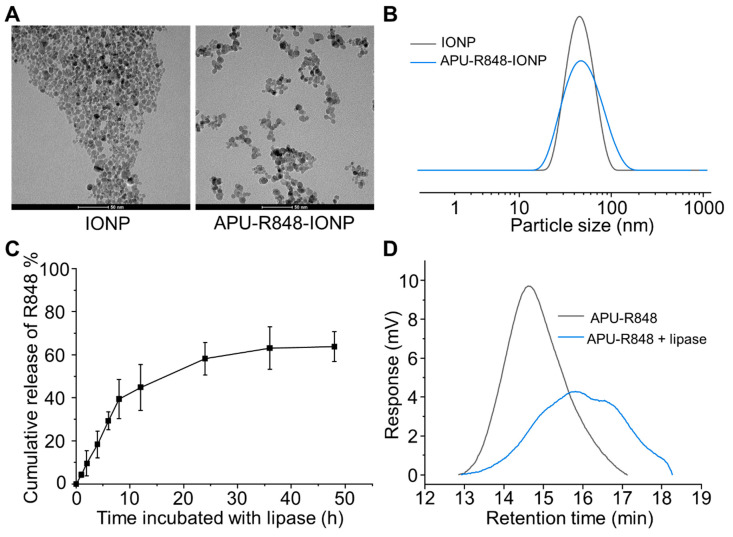
Characterization of APU-R848-IONPs. (**A**) TEM morphology of nanoparticles. Scale bar = 50 nm. (**B**) Hydrodynamic size and distribution of IONPs without polymer coating and APU-R848-IONPs. (**C**) Cumulative release of R848 from APU-R848-IONPs in lipase solution (3 mg/mL) for 48 h. (**D**) Molecular weight characterization of APU-R848 before and after incubation with lipase.

The morphology of the APU-R848-IONPs was characterized with TEM and shown in Figure 2A. The TEM morphology of the IONPs without a polymer coating indicated a rather aggregated morphology. In contrast, the APU-R848-IONPs showed a significantly improved dispersion, with isolated nanoparticles, demonstrating that the polymer coating mitigates aggregation.

The hydrodynamic diameter increased from 37.5 ± 8.2 nm (IONPs without polymer coating) to 58.5 ± 6.1 nm (APU-R848-IONPs) (Table 1 and Figure 2B). Despite minor residual clustering observed in TEM due to sample drying artifacts, the increase in hydrodynamic size reflects the physical thickness of the polymer coating and its associated hydration layer. The IONPs without a polymer coating exhibited a negative surface charge of −30.5 ± 7.2 mV, attributed to DMSA capping. After being coated with APU or APU-R848, IONPs exhibited slightly cationic surface charges due to the presence of arginine moieties in APU. APU-R848-IONPs exhibited a zeta potential of 5.6 ± 2.3 mV, whereas APU-IONPs demonstrated a zeta potential of 15.2 ± 5.5 mV. The cationic surface charge originates from the arginine moieties present in APU and APU-R848. Compared to APU, the cationic charge was partially neutralized by the 4-MBA modification and the complexation with R848. As a result, a slightly reduced cationic charge was detected in APU-R848-IONPs.

To characterize the R848 loading content in APU-R848-IONPs, the polymer coating was digested in an acidic alcohol solution, and the fully liberated R848 in the supernatant was analyzed using HPLC. The loading content of R848 was determined as 20.5 ± 3.8% relative to the total polymer content. Furthermore, the degradation of APU-R848 and the enzyme-triggered release of R848 from APU-R848-IONP were characterized. The APU polymer contained ester groups that were susceptible to degradation triggered by lipase. The cleavage of the polymeric structure could result in the release of R848 molecules. The APU-R848-IONPs were incubated with lipase (1 mg/mL) for 48 h, and the concentration of R848 in the supernatants was characterized via HPLC. The lipase-triggered release of R848 release is shown in Figure 2C. An accumulative release of over 60% of the total R848 was detected after 48 h incubation with lipase. The R848 loading content in APU-R848-IONPs was quantified via complete digestion of the polymer coating in an acidic alcohol solution (70% nitric acid: 36% hydrochloric acid, 1:1 *v*/*v*), which liberates the encapsulated R848 for HPLC analysis. This method measured the total payload capacity of the nanoparticles. In contrast, the enzyme-triggered release profile was assessed under physiologically relevant conditions (pH 7.4, lipase-containing buffer) to simulate biodegradation in vivo. The release can be correlated with the decrease in molecular weight detected by GPC (Figure 2D). The molecular weight of APU-R848 decreased from 26,765 Da to 7516 Da after 48 h incubation with lipase. The cleavage and dissociation of polymers resulted in the release of R848.

### 3.4. Internalization of APU-R848-IONPs and the Re-Polarization Effect in Macrophages In Vitro

**Figure 3:**
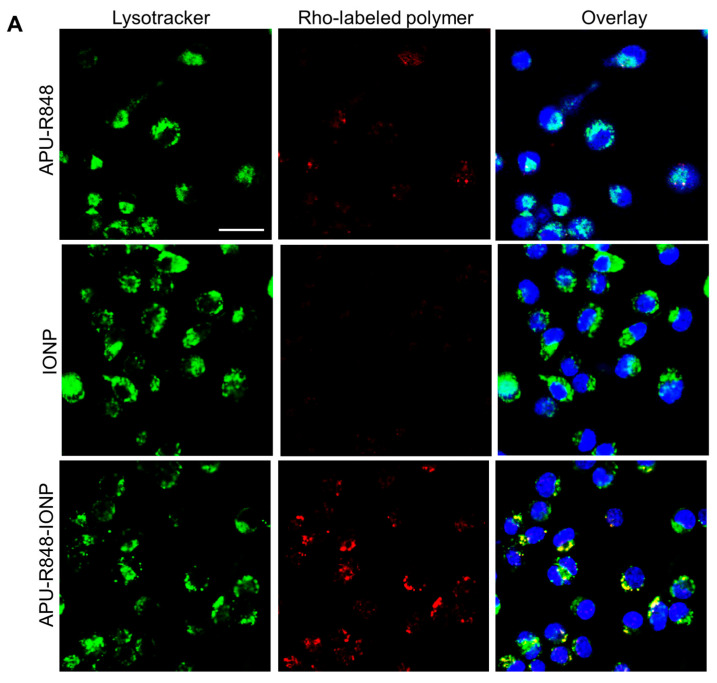

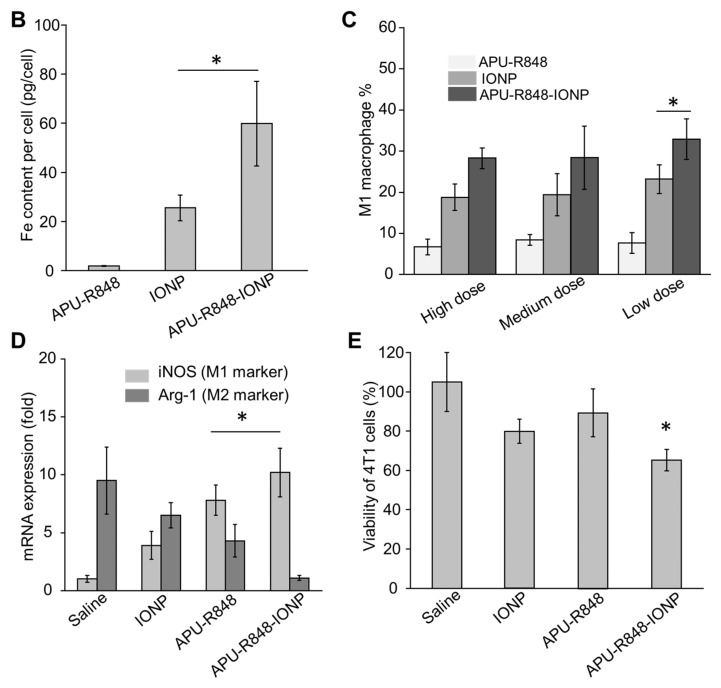
In vitro study of APU-R848-IONPs in BMDMs. (**A**) Visualization of the uptake of APU-R848-IONPs, IONPs alone or APU-R848 alone after incubation with BMDMs for 4 h. The APU-R848 polymer was labeled with rhodamine, while the macrophages were stained with Lysotracker Green. The intracellular fluorescence was visualized by confocal microscopy (scale bar = 50 µm). (**B**) The intracellular Fe content in BMDMs after 4 h incubation was detected by ICP-OES (n = 3). (**C**) Detection of macrophage polarization in BMDMs. Nanoparticles with different APU-R848 to IONP ratios were incubated with BMDMs for 48 h, and the percentage of M1 macrophages was analyzed via flow cytometry (n = 3). (**D**) Detection of characteristic mRNA expression. Macrophages treated with various nanoparticles were assessed for the mRNA expression of iNOS (M1) and Arg-1 (M2) via QT-PCR (n = 3). (**E**) Transwell co-culture of 4T1 cells (in the lower compartment) and macrophages (in the upper compartment) pre-treated with APU-R848-IONPs. The viability of 4T1 cells after 48 h was determined (n = 3). Statistical significance: *, *p* < 0.05.

We first compared the cytotoxicity of APU-R848-IONPs in RAW264.7 macrophages. As shown in Figure S6 (Supplementary Materials), biocompatibility was observed across the tested range of 25 to 300 μg Fe/mL. Compared to IONPs alone (without a polymer coating) or APU-R848 alone, no significant toxicity was observed with APU-R848-IONPs. The endocytosis of APU-R848-IONPs was then explored in bone marrow-derived macrophages (BMDMs). Firstly, rhodamine-labeled APU-R848 was coated onto IONPs and incubated with BMDMs for 4 h. The intracellular fluorescence was characterized by confocal microscopy. As shown in Figure 3A, no significant red fluorescence was detected in rhodamine-labeled APU-R848-treated BMDMs, which could be attributed to the absence of nanostructures. The APU-R848 polymer exhibited a good aqueous dispersibility; however, it did not form a nano-assembled structure. Nevertheless, the internalization of particles with micron- and nanometer sizes was favorable in macrophages [39]. In contrast, significant red fluorescence was observed in BMDMs treated with APU-R848-IONPs, which co-localized with Lysotracker fluorescence (Figure 3A), indicating the efficient internalization of APU-R848-IONPs by BMDMs. Supplementary to the confocal study, the intracellular Fe content of BMDMs incubated with APU-R848 alone, IONPs alone or APU-R848-IONPs for 4 h was determined by ICP-OES. As shown in Figure 3B, a 2.7-fold increase in intracellular Fe content was detected in macrophages treated with APU-R848-IONPs compared to IONPs alone, which could be related to the slightly positive surface charge of APU-R848-IONPs. The results indicated that APU-R848-IONPs effectively facilitated the internalization of both R848 and IONPs by macrophages.

The re-polarization of macrophages was subsequently investigated. BMDMs were first differentiated into M2 macrophages by 10 ng/mL IL-4 for 24 h, followed by incubation with APU-R848-IONPs at different doses for an additional 24 h (high dose: 100 μg Fe/mL; medium dose: 50 μg Fe/mL; low dose: 25 μg Fe/mL). Figure 3C indicates the percentage of CD80^+^ cells (M1 marker) detected by flow cytometry. APU-R848-IONPs, at all three doses, effectively converted the M2 population into M1 macrophages. APU-R848-IONPs at a low dose of 25 μg Fe/mL resulted in a re-polarized M1 population of 32.9 ± 4.9%, which was significantly greater than that observed with IONPs alone or APU-R848 alone. To further consolidate the M1/M2 re-polarization effect in macrophages, a QT-PCR study was conducted to assess the expression levels of representative M1 and M2 mRNA. Figure 3D compares the mRNA expression of iNOS (M1 marker) and Arg-1 (M2 marker) in BMDMs treated with APU-R848-IONPs for 24 h. A significantly higher expression of Arg-1 over iNOS was detected in saline-treated macrophages. On the contrary, a reversed expression of iNOS over Arg-1 was observed in APU-R848-IONP-treated macrophages. This elevation in iNOS mRNA levels was significant compared to IONPs alone or APU-R848 alone. APU-R848-IONPs delivered R848 (a TLR7/8 agonist) and IONPs (a ROS inducer) to macrophages, which could trigger dual iNOS activation via NF-κB and ROS pathways [40]. APU-R848 alone (polymer–drug complex) showed limited uptake (Figure 3A), reducing R848 bioavailability and iNOS induction (Figure 3D). IONPs alone could also be challenged by an insufficient bioavailability, diminishing cellular interaction and ROS signaling. Therefore, the elevation in iNOS mRNA levels induced by APU-R848-IONPs was significant compared to IONPs alone or APU-R848 alone.

APU-R848-IONP-treated macrophages were then co-cultured with 4T1 breast cancer cells for 48 h. As shown in Figure 3E, the inhibition of 4T1 cell proliferation was more pronounced when macrophages seeded on the upper compartment of the Transwell were pre-treated with APU-R848, as compared to APU-R848 or IONPs alone. The APU-R848-IONPs consisted of both APU-R848 moieties and the IONP core, with a regulatory effect on macrophages, which provided the potential to activate anti-tumor immunity. The tumor microenvironment comprises various cell types, including macrophages, T cells and other immune cells. Hurdles exist in in vitro cell culture to mimic the complex microenvironment of tumors. Therefore, the following study was performed on a murine 4T1 TNBC model to investigate anti-tumor effects and immune regulation.

### 3.5. APU-R848-IONPs Demonstrated Significant Tumor Inhibition in 4T1 Model

**Figure 4:**
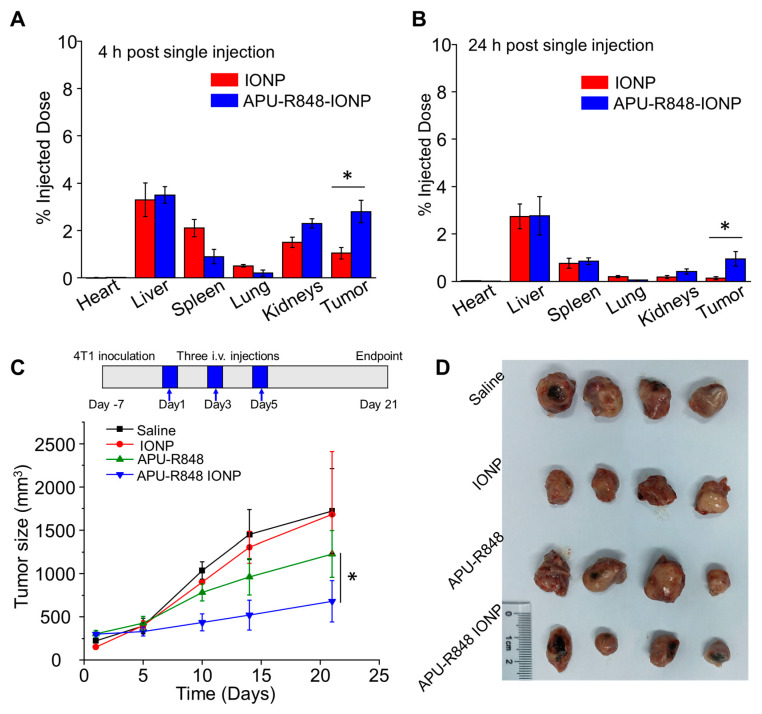

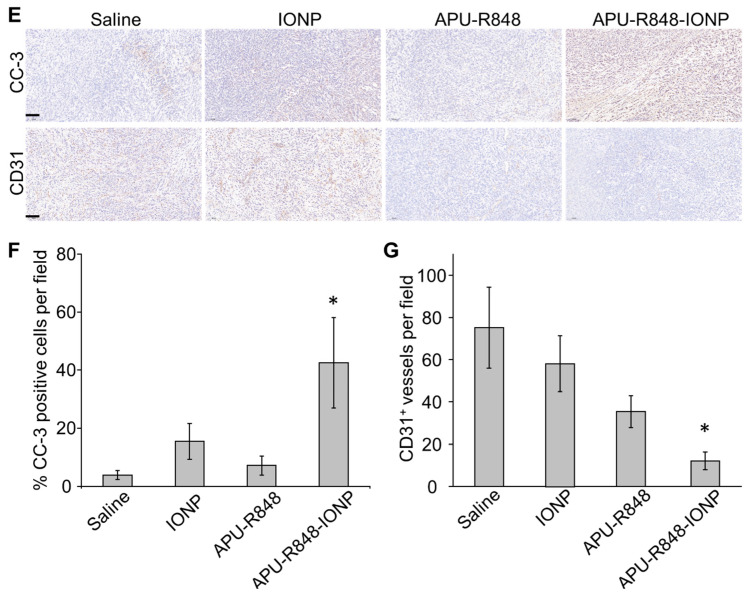
In vivo biodistribution and anti-tumor study of APU-R848-IONPs in a 4T1 orthotopic breast tumor model in mice. (**A**,**B**) Biodistribution study. 4T1 tumor-bearing mice received a single intravenous injection of APU-R848-IONPs (10 mg Fe/kg). At 4 h or 24 h post injection, the tumor tissue and major organs were collected and homogenized, and the Fe content was detected via ICP-OES. The normalized percentage of Fe content relative to the total injected dose was plotted. (**C**,**D**) 4T1 tumor-bearing mice received intravenous injections of saline, APU-R848 alone, IONPs alone (without a polymer coating) or APU-R848-IONPs (n = 4). Three intravenous injections were administered on days 1, 3 and 5 (5 mg Fe/kg/injection). The tumor growth and the images of tumor collected on day 21 were recorded. (**E**) The expression of cleaved caspase-3 (CC-3) and CD31 in tumor sections collected at the endpoint (day 21) was detected by immunohistochemical (IHC) staining, scale bar = 100 μm. (**F**) The percentage of cleaved caspase-3 positive cells was quantified to characterize apoptosis. (**G**) The number of CD31-positive blood vessels per unit area was compared to characterize the anti-angiogenesis effect (scale bar = 100 μm). Five ROIs were evaluated for each IHC image analysis. Statistical significance: *, *p* < 0.05.

A biodistribution study was performed on the murine 4T1 TNBC model. At 4 h or 24 h post the injection of APU-R848-IONPs, the Fe concentrations in major organs and tumors were detected via ICP-OES, normalized to the total injected dose and plotted in Figure 4A,B. Compared to IONPs alone (without a polymer coating), APU-R848- IONPs at 4 h demonstrated a 2.7-fold increase in intratumoral Fe concentration. At 24 h, APU-R848-IONPs exhibited a 7.3-fold increased Fe concentration in the tumor compared to IONPs alone. The APU-R848 coating can provide the colloidal stabilization for IONPs to prevent IONPs’ aggregation (Figure 2A) via steric hindrance, enabling systemic circulation and enhancing the accumulation of IONPs at the tumor site. In addition, the nanoparticle size (58.5 nm) exploits the enhanced permeability and retention effect [41], which can promote vascular extravasation and intratumoral retention. Reduced liver/spleen accumulation of APU-R848-IONPs (Figure 4B) confirmed the evasion of the reticuloendothelial system (RES).

The anti-tumor effect of APU-R848-IONPs was demonstrated in a murine orthotopic 4T1 breast tumor model. The mice received intravenous injections of APU-R848-IONPs, and the size of the 4T1 breast tumors was monitored for 21 days post injection. The measurements of tumor size and the tumor inhibitory effect are plotted in Figure 4C,D. A significant anti-tumor effect was observed in the APU-R848-IONP group (506.5 ± 98.2 mm^3^) compared to APU-R848 alone (1025.6 ± 132.5 mm^3^), IONPs alone (1506.3 ± 162.2 mm^3^) or the saline group (1623.2 ± 245.2 mm^3^) on day 21. In addition to monitoring tumor growth, the survival of 4T1 tumor-bearing mice receiving various treatments was assessed, as shown in Figure S7 (Supplementary Materials). Mice in the APU-R848-IONP treatment group demonstrated an 80% survival rate by day 35. In contrast, the survival rate of mice treated with APU-R848 decreased to 50%. In the saline- and IONP-treated groups, all mice were euthanized due to the excessive tumor burden.

The tumor inhibitory effect was further investigated through analyses of apoptosis and anti-angiogenesis. An immunohistochemistry (IHC) was conducted to detect cleaved caspase-3, which functioned as a representative biomarker for apoptosis. Figure 4E shows a significant abundance of cleaved caspase-3 (CC-3)-positive cells (indicated by brown pixels) in tumors treated with APU-R848-IONPs on day 21. Quantitative analysis of CC-3 positivity (Figure 4F) indicated that APU-R848-IONPs were significantly more effective in inducing apoptosis at the tumor site compared to IONPs alone, APU-R848 alone or saline. The CD31 staining of micro-blood vessels in tumor sections was subsequently evaluated. Intact blood vessels were observed in tumor sections treated with both saline and IONPs alone (Figure 4E). A significantly reduced blood vessel density (12.1 ± 4.2 vessels/field) in the tumor tissue treated with APU-R848-IONPs is evident in Figure 4G, and the anti-angiogenesis effect was more efficient compared to APU-R848 alone (35.3 ± 7.5 vessels/field), IONPs alone (58.2 ± 13.3 vessels/field) or saline (75.2 ± 19.2 vessels/field).

The results indicated that APU-R848-IONPs inhibited the formation of blood vessels in tumor tissues and induced apoptosis, collectively contributing to its anti-tumor efficacy. Compared to IONPs alone or APU-R848 alone, the enhanced efficacy of APU-R848-IONPs in inhibiting 4T1 tumors can be attributed to its improved biodistribution profile and the modulation of the tumor microenvironment.

### 3.6. APU-R848-IONPs Regulated the Tumor Microenvironment and Activated Immune Surveillance in 4T1 Tumors

**Figure 5:**
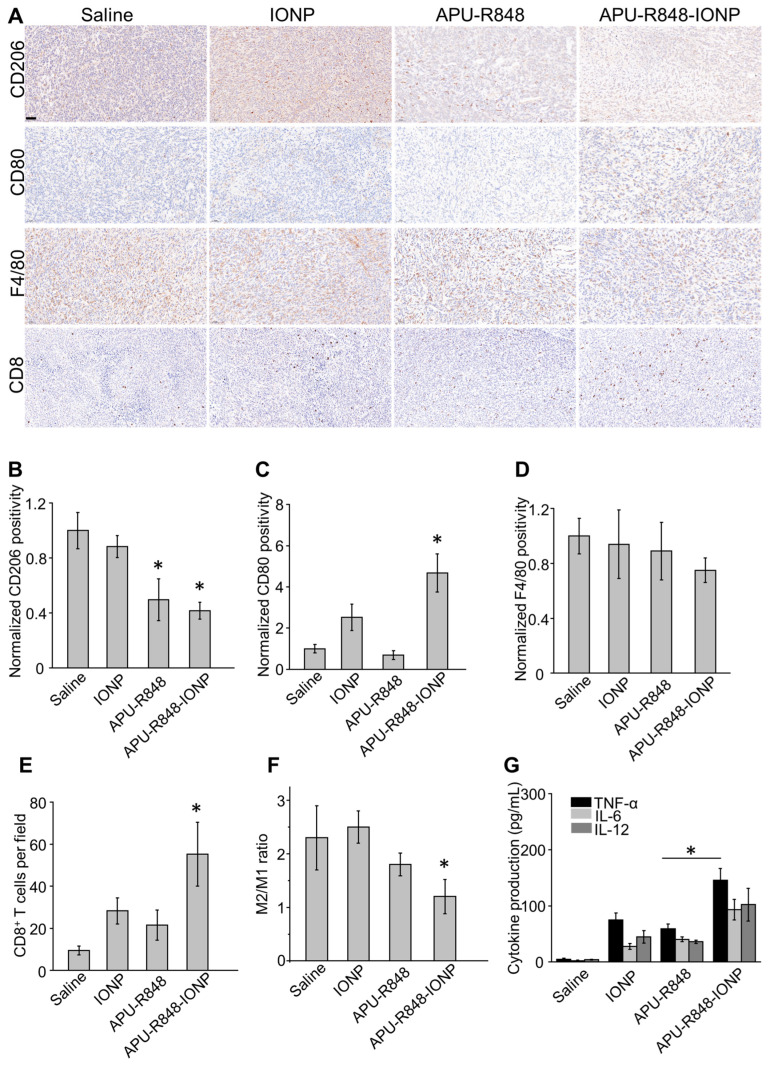
APU-R848-IONPs activated anti-tumor immunity in 4T1 tumors. (**A**–**E**) Immunohistochemistry staining of 4T1 tumor tissues (collected on day 7) to characterize the expression of CD206, CD86, F4/80 and CD8, including representative images and quantifications (scale bar = 50 µm). (**F**) M2/M1 macrophage ratio (ratio of CD206^+^ cells to CD80^+^ cells within the gated F4/80^+^ cell population) in tumors receiving different treatments. (**G**) ELISA detection of tumoricidal cytokine production in 4T1 tumors (n = 3). Statistical significance: *, *p* < 0.05.

Macrophages in the 4T1 tumor microenvironment have been reported to exhibit an immune-tolerant state [5], which acts as a major barrier to anti-tumor immunity. Therefore, the activation of immune surveillance against tumor cells could be crucial for the therapeutic efficacy of APU-R848-IONPs. Tumors harvested at day 7 revealed early immune microenvironment remodeling, which preceded tumor regression (Figure 4C). To elucidate the immune responses associated with treatment efficacy, an immunohistochemistry analysis was conducted on 4T1 tumors collected on day 7 following the initial injection of APU-R848-IONPs (Figure 5A). A general biomarker for mature macrophages (F4/80) and representative biomarkers for M1 macrophages (CD80) and M2 macrophages (CD206) were analyzed to characterize the in vivo regulatory effect of APU-R848-IONPs on tumor-associated macrophages (TAMs). The representative IHC images (Figure 5A) and quantification (Figure 5B) suggested an overexpression of CD206 in saline-treated 4T1 tumors, suggesting an abundance of M2 macrophages that played a significant role in the immunosuppressive microenvironment. APU-R848-IONP treatment significantly reduced CD206 expression, while stimulating CD80 expression (Figure 5B,C). However, the regulatory effect of APU-R848-IONPs on total F4/80 expression was not significant (Figure 5D). To further investigate the trend, 4T1 tumors were collected, and the macrophage population was quantitatively analyzed via flow cytometry. In Figure 5F, a significant decrease in F4/80^+^CD206^+^ macrophages (M2 phenotype) and a notable increase in F4/80^+^CD80^+^ macrophages (M1 phenotype) are detected, as compared to the APU-R848 alone, IONP alone or saline-treated groups. The results indicated that APU-R848-IONPs could significantly stimulate the suppressive tumor microenvironment by re-polarizing TAMs into the M1 phenotype, which was consistent with the in vitro macrophage re-polarizing effect (Figure 3C,D). In addition to macrophage polarization in vivo, a significantly higher frequency of CD8^+^ T cells was detected in 4T1 tumors treated with APU-R848-IONPs (Figure 5A,E) compared to the other groups. The elevated level of CD8^+^ cytotoxic T cells and pro-inflammatory macrophages could collectively enhance the activation of effective anti-tumor immune responses [42].

The anti-tumor immune regulatory potential of APU-R848-IONPs was further demonstrated by the detection of representative pro-inflammatory cytokines. A significantly increased production of TNF-α, interleukin-6 (IL-6) and interleukin-12 (IL-12) was detected in 4T1 tumors treated with APU-R848-IONPs (Figure 5G). The induction of pro-inflammatory cytokines by APU-R848-IONPs was significantly greater compared to the IONP alone, APU-R848 alone or saline-treated groups. The representative pro-inflammatory cytokines (TNF-α, IL-6 and IL-12) are critical to a tumoricidal effect and play key roles in inhibiting tumor cells [35,43]. The inhibitory effect of APU-R848-IONPs on 4T1 TNBC (Figure 4C) showed a strong correlation with the activation of the immune response and the production of tumoricidal pro-inflammatory cytokines.

### 3.7. Biosafety Evaluation of APU-R848-IONP Treatment

**Figure 6:**
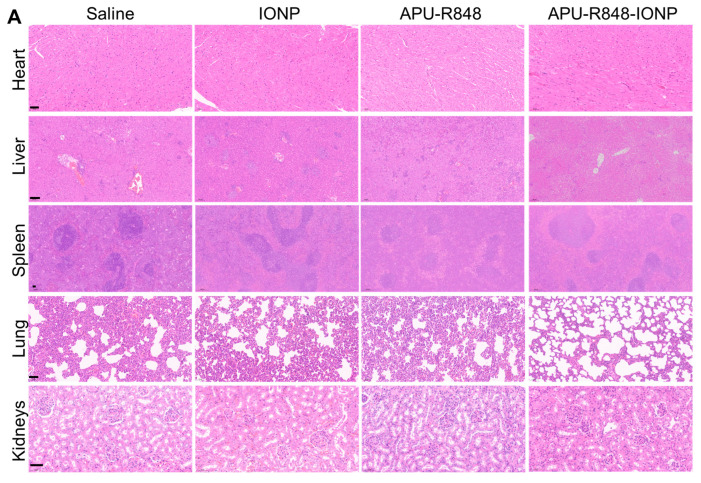

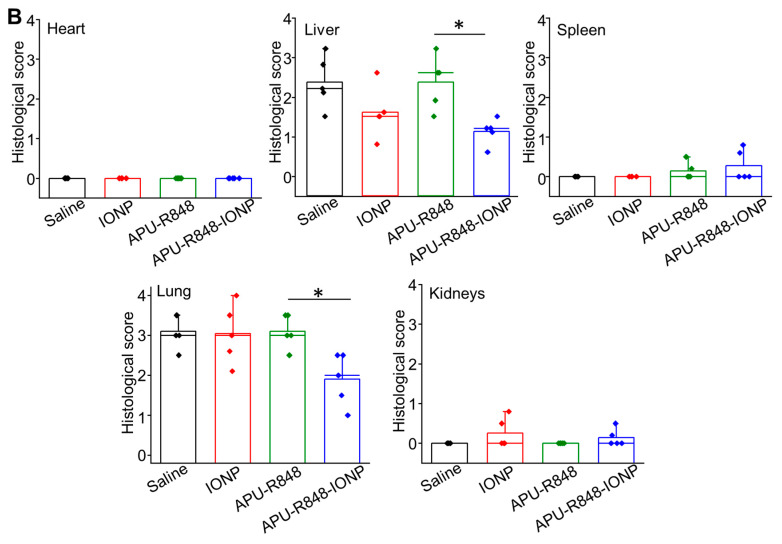
Histological analysis of major organs from 4T1 tumor-bearing mice collected on day 21. (**A**) Representative H&E staining of major organs including heart, liver, spleen, lung and kidneys. Scale bar = 50 μm. (**B**) Histological score of the H&E staining. Five ROIs were analyzed for each group, and the quantitative scores are expressed as mean ± SD. Statistical significance: *, *p* < 0.05.

For the histological examination, H&E staining was performed on sections of major organs from 4T1 tumor-bearing mice collected on day 21 (Figure 6A). No abnormalities were observed in the heart, kidneys or spleen of mice receiving APU-R848-IONPs. The quantitative analysis presented in Figure 6B demonstrates that the histological scores in the APU-R848-IONP group were equivalent to those in the saline control group, indicating a lack of organ toxicity. The abnormalities in lung tissues observed in groups treated with saline, IONPs alone or APU-R848 alone correlated with the metastatic characteristics of the 4T1 breast tumor to lung tissue [44]. The reduction in histological scores in lung observed with APU-R848-IONPs was positively correlated with an enhanced anti-tumor effect. In addition to lung toxicity, IONP-alone-treated mice exhibited histopathological effects on the liver, including hepatocyte necrosis and lymphocytic infiltration. Except for the APU-R848-IONP-treated group, focal areas of extramedullary hematopoiesis were [45] observed in the livers from 4T1 tumor-bearing mice in all the groups, indicating potential liver metastasis. The hepatic abnormalities in the group treated with IONPs alone could be attributed to their accumulation following intravenous injection and the prolonged disruption of liver iron homeostasis [46]. The significantly reduced liver toxicity of APU-R848-IONPs (Figure 6B) was correlated with the surface coating of IONPs by APU-R848 polymer, which could shield the nanoparticles from direct interaction with liver tissue.

While APU-R848-IONPs demonstrated significant promise in re-polarizing macrophages and enhancing anti-tumor immunity in TNBC, several limitations warrant consideration for clinical translation. First, concerns regarding long-term toxicity could persist. Despite the APU-R848 coating mitigating acute liver toxicity compared to IONPs alone (Figure 6), the potential accumulation of iron in reticuloendothelial organs (e.g., liver, spleen) following repeated systemic administration could chronically disrupt iron homeostasis and induce oxidative stress [47,48]. Comprehensive chronic toxicity studies evaluating iron biodistribution, organ function and oxidative damage over extended periods are essential. Second, the scalability of the nano-platform could face challenges. The synthesis of the functionalized APU-R848 polymer involved multi-step chemistry (thiol-ene click reaction, R848 complexation) and a precise optimization of the coating (Figure S5, Supplementary Materials), which may complicate reproducible large-scale manufacturing. Streamlining polymer synthesis and nanoparticle formulation processes is crucial for clinical feasibility. Third, systemic immune activation by APU-R848-IONPs could raise the risk of immune-related adverse events, such as uncontrolled cytokine release or autoimmune responses [49], as evidenced by elevated pro-inflammatory cytokines (Figure 5G). A careful dose optimization and the development of tumor-targeted delivery strategies (e.g., hybrid coatings with tumor-specific ligands) are critical to minimize systemic exposure and off-target immunogenicity [28]. Future work should focus on these areas to enhance the safety profile and translational potential of APU-R848-IONPs.

## 4. Conclusions

This study developed an immune-regulatory nano-platform by engineering iron oxide nanoparticles coated with arginine-based pseudo-protein conjugates complexed to R848 (APU-R848-IONPs), demonstrating the potential for TNBC immunotherapy. The multifunctional APU-R848 conjugate integrated an arginine-containing pseudo-protein with a TLR7/8 agonist (R848) via electrostatic and π-π interactions. This conjugate provided a dual function: (1) as a stable and functional coating for IONPs that significantly improved colloidal stability and tumor biodistribution (demonstrated by a 7.3-fold increase in the accumulation of intratumoral Fe compared to IONPs alone) and (2) as a therapeutic payload delivery system exploiting synergistic immunomodulation. The APU-R848-IONP platform reprogrammed tumor-associated macrophages by coupling R848-driven and IONP-mediated regulation, reducing the M2/M1 macrophage ratio by 51% and elevating tumoricidal cytokines (TNF-α, IL-12). This reshaped microenvironment further recruited CD8^+^ T cells and induced apoptosis, collectively amplifying anti-tumor immunity to achieve a 3.2-fold increase in anti-tumor efficacy compared to the control. The APU-R848-IONP platform represents a significant advancement by integrating a targeted nanomaterial delivery with synergistic immunomodulation. APU-R848-IONPs effectively overcome immunosuppression by re-polarizing macrophages, activating robust immune responses and offering a promising new modality for TNBC immunotherapy with a favorable biosafety profile.

## Figures and Tables

**Table 1:** Characterization of size and zeta potential for APU-R848-IONPs.

	Zeta Potential in Saline	DLS Size	TEM Size
IONP (without polymer coating)	−30.5 ± 7.2 mV	37.5 ± 8.2 nm	12.5 ± 3.6 nm
APU-IONP (IONP coated with APU)	15.2 ± 5.5 mV	50.6 ± 7.5 nm	n.a.
APU-R848-IONP (IONP coated with APU-R848)	5.6 ± 2.3 mV	58.5 ± 6.1 nm	13.2 ± 3.0 nm

## Data Availability

The raw data supporting the conclusions of this article will be made available by the authors on request.

## Supplementary Materials

The following supporting information can be downloaded at: https://www.mdpi.com/article/10.3390/nano15131006/s1, Figure S1. Structural features in amino-acid-based poly(urea urethane), abbreviated as APU. Figure S2. Synthesis of R848-complexed APU polymer (abbreviated as APU-R848). Figure S3. ^1^H-NMR characterization of 4-MBA-modified APU (abbreviated as 4-MBA-APU). (A) Comparison of ^1^H-NMR spectra of APU before 4-MBA functionalization. (B) Peak attribution in 4-MBA-APU. Figure S4. Molecular weight characterization of the 4-MBA-APU by gel permeation chromatography (GPC). Figure S5. Tuning of APU-R848 polymer structures and coating procedures to develop optimized APU-R848-coated IONPs. Figure S6. Cytotoxicity study of APU-R848-IONPs in RAW264.7 macrophages. Figure S7. Survival rate of 4T1-bearing mice receiving intravenous injections of saline, APU-R848 polymer, uncoated IONPs or APU-R848-IONPs (n = 4). Refs. [14,50] are cited in the supplementary materials.
