# Supplementary material for: Development of Immune-Regulatory Pseudo-Protein-Coated Iron Oxide Nanoparticles for Enhanced Treatment of Triple-Negative Breast Tumor
Source: Nanomaterials (Basel). 2025 Jun 30;15(13):1006. doi: 10.3390/nano15131006 (PMC12250821; doi:10.3390/nano15131006)
Supplement: Supplementary file 1 [file nanomaterials-15-01006-s001.zip › nanomaterials-3673707-supplementary.pdf]

Supplementary Materials

Development of immune-regulatory pseudo-protein-coated iron oxide nanoparticles for enhanced treatment of triple-negative breast tumor

Ying Ji[a*], Juan Li[b*], Li Ma[a,c], Zhijie Wang[b], Bochu Du[a], Hiu Yee Kwan[d], Zhaoxiang Bian[d], Chih-Chang Chu[e]

[a] Research Institute for Intelligent Wearable Systems, Hong Kong Polytechnic University, Hong Kong SAR, PR China.

[b] CAS Key Laboratory for Biomedical Effects of Nanomaterial & Nanosafety, Institute of High Energy Physics, Chinese Academy of Sciences, Beijing 100049, PR China.

[c] College of Biomass Science and Engineering, Sichuan University, Chengdu 610065, PR China.

[d] School of Chinese Medicine, Hong Kong Baptist University, Hong Kong SAR, PR China.

[e] Biomedical Engineering Field, Cornell University, Ithaca, NY, 14853, United States.

* Co-corresponding authors. E-mail address: ying.ji@polyu.edu.hk (Y. Ji) and lijuan@ihep.ac.cn (J. Li)

Synthesis of APU polymer

Arginine-based alkylene diester monomers were synthesized according to previous research[1]. L-arginine (L-Arg) hydrochloride (0.04 mol) and 1,4-butanediol (0.02 mol) were directly condensed in refluxed toluene (80 mL) with the presence of p-toluenesulfonic acid monohydrate (0.05 mol). The heterogeneous solid-liquid reaction mixture was heated to 120 °C and refluxed for 48 h after 1.62 mL (0.09 mol) of water was generated and collected by a dean-stark apparatus. After the reaction was ended and cooled to room temperature, toluene was decanted. The resulting monomer was purified by recrystallization for three times in cold isopropyl alcohol (-20 °C) and then was dissolved in 50 mL of deionized water. The toluenesulfonic acid ion was then neutralized by 2 M sodium bicarbonate to reach a final pH of 9.5. The resulting aqueous solution of bis (L-Arg) butane diester was lyophilized.

APU was synthesized using a two-step solution polymerization[1, 2]. In the first step, 8.41 g hexamethylene diisocyanate (HDI, 0.05 mol) in 30 mL DMSO was stirred continuously with 3.30 g glycerol α-monoallyl ether (GAE, 0.025 mol) in 20 mL DMSO. Stannous 2-ethyl-hexanoate catalyst (2 wt% of total mass of reactants), and 2, 6 di-tert-butyl-4-methyl phenol (1 wt% of total mass of reactants) were added to the HDI/GAE mixture. 2, 6 di-tertbutyl-4-methyl phenol acted as an antioxidant to protect the double bond of GAE. The mixture was maintained at 60 °C for 5 h under dry nitrogen atmosphere. In the second step, a solution of Arg-2-Cl monomer (0.025 mol in 80 mL DMSO) was added to the prepolymer solution, and the reaction was maintained at 60 °C for another 18 h under dry nitrogen atmosphere. The resulting polymer was precipitated in ethyl acetate to remove residual chemicals like stannous 2-ethyl-hexanoate, 2, 6 di-tert-butyl-4-methyl phenol, DMSO and unreacted monomers. Then, the precipitant was vacuum dried at room temperature first, followed by re-dissolving in DMF. The polymer in DMF solution was repetitively precipitated in deionized water before vacuum drying.

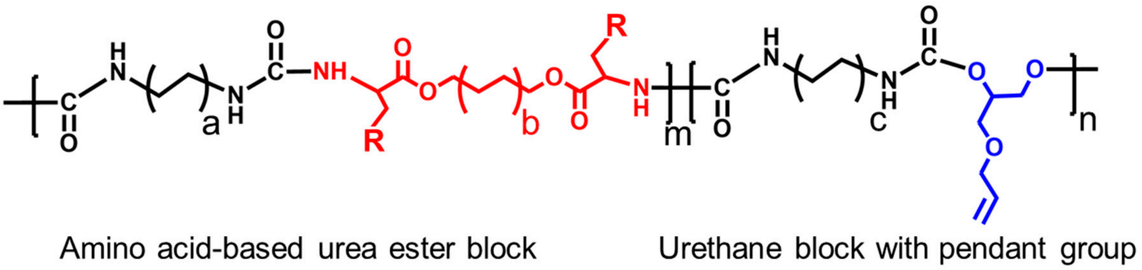

Figure S1. Structural features in amino-acid-based poly(urea urethane), abbreviated as APU.

Synthesis of APU-R848 polymer

The double-bond and other functional pendant groups of APU polymer (Figure S1) can be functionalized to form complexation with anti-tumor therapeutic molecules and to develop functional coatings for metal oxide nanoparticles. One example was given below for the functionalization of APU polymer (Figure S2).

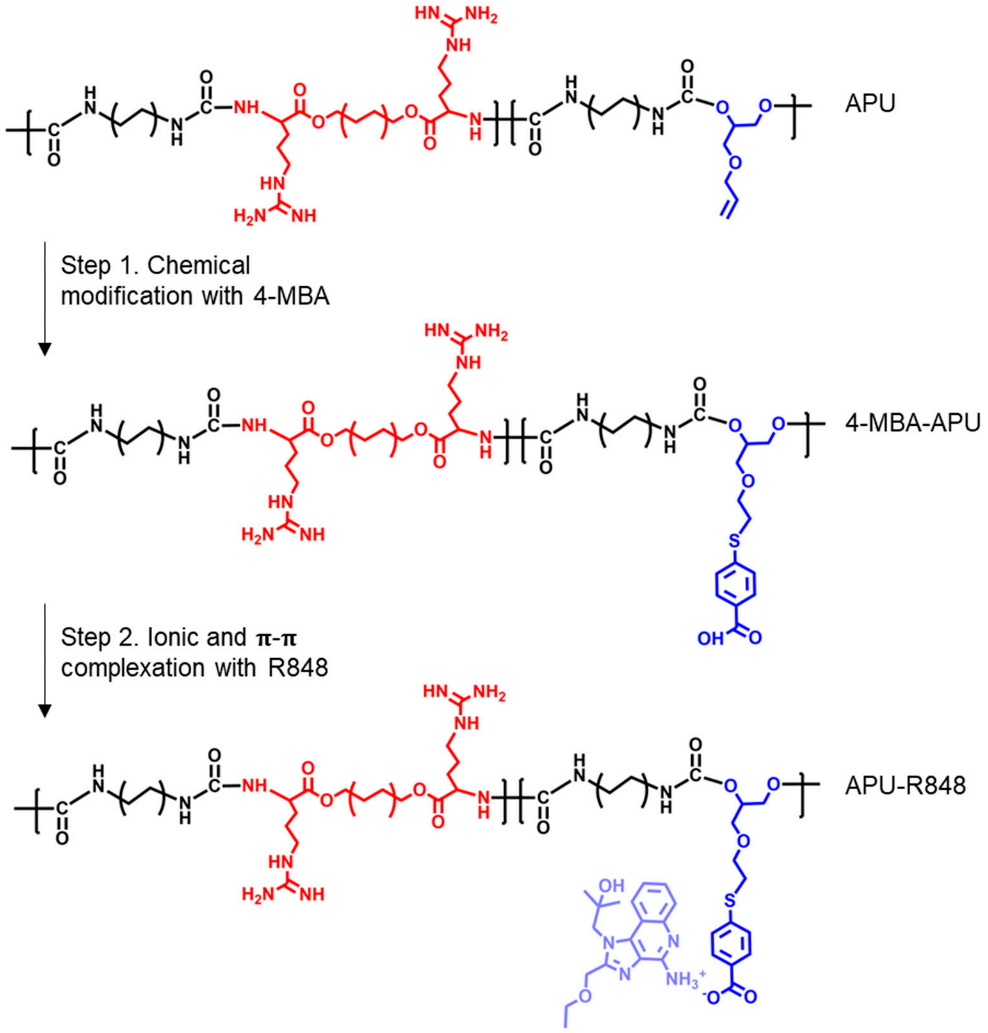

Figure S2. Synthesis of R848-complexed APU polymer (abbreviated as APU-R848).

In the first step, modification of APU was performed via thiol-ene click chemistry between the allyl groups in APU and the thiol groups in 4-mercaptobenzoic acid (4-MBA). 1 mmol of 4-MBA, 1.5 mmol of azobisisobutyronitrile (AIBN) and 0.85 g of APU were dissolved in 50 mL of DMF. The solution was degassed and purged with nitrogen. The reaction was maintained

at 65°C for 24 h. The resulting polymer was recovered and purified by repetitive precipitation in ethyl acetate to remove unreacted 4-MBA and AIBN. The resulting 4-MBA-APU (Figure S2) is an intermediate polymer that could form electrostatic interaction or π–π interaction with therapeutic molecules.

In the second step, R848 (Resiquimod), as an agonist of the toll-like receptors 7 and 8 (TLR7 and TLR8) was then conjugated to 4-MBA-APU. The weakly basic amine groups of R848 could form electrostatic interaction with carboxylates in 4-MBA. Furthermore, the fused cyclic hydrocarbons in R848 could form π–π interaction with 4-MBA as well.

To form a complexation between R848 and 4-MBA-APU, 1 g of 4-MBA-APU polymer was dissolved in 10 mL DMSO under stirring (500 rpm) at room temperature. R848 was dissolved in DMSO and added dropwise into the polymer solution. The feed ratio between 4-MBA-APU and R848 was tunable from 0.01: 1 to 0.25:1 (w/w). The resulting R848-complexed APU (R848-APU) was repetitively precipitated in excessive ethanol to remove any free R848. The precipitate was dried in vacuo and stored at -20 °C for further use.

Characterization of the as-developed 4-MBA-APU and APU-R848 polymers

Firstly, the chemical structure of the 4-MBA-APU was characterized by ^{1}H-NMR in *d6*-DMSO as shown in Figure S3A. After modification, APU lost vinyl peaks at the chemical shift from 5 to 6 ppm, which indicated the consumption of double bonds by the thiol-ene reaction with 4-MBA (Figure S3A). New peaks at 7 to 8 ppm from phenyl groups (from 4-MBA) appeared in 4-MBA-APU, which demonstrated the chemical conjugation. The detailed peak attribution of the ^{1}H-NMR spectra of 4-MBA-APU was shown in Figure S3B. The weight average molecular weight of 4-MBA-APU was detected as 22350 Da, with PDI=3.22. The weight average molecular weight of APU-R848 was detected as 26765 Da, with PDI=2.59 (Figure S4).

A

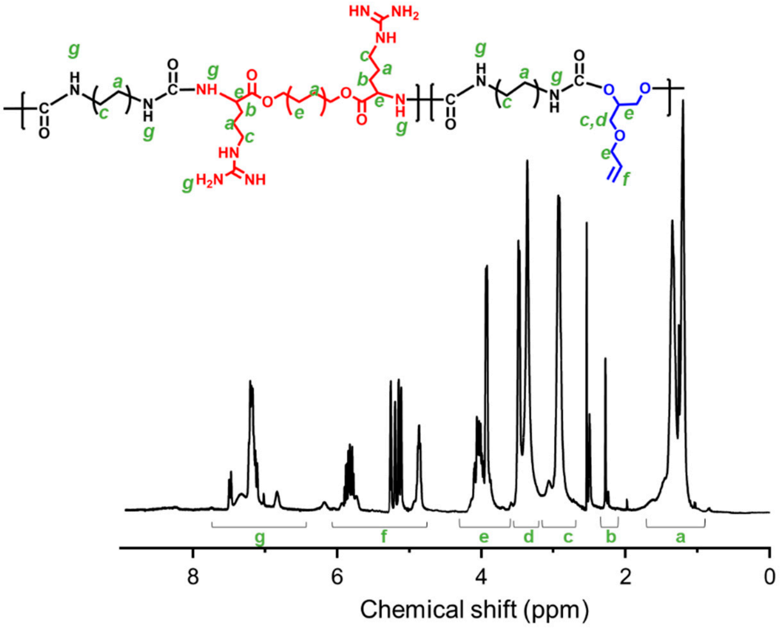

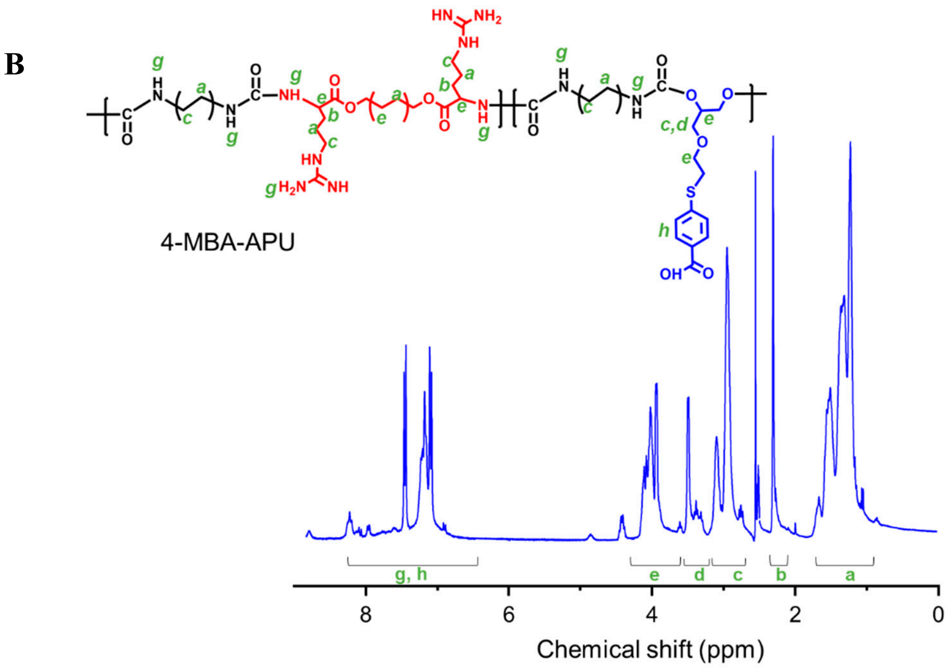

Figure S3. ^{1}H-NMR characterization of the 4-MBA modified APU (abbreviated as 4-MBA-APU). (A) The comparison of ^{1}H-NMR spectra of APU before 4-MBA functionalization. (B) Peak attribution in 4-MBA-APU.

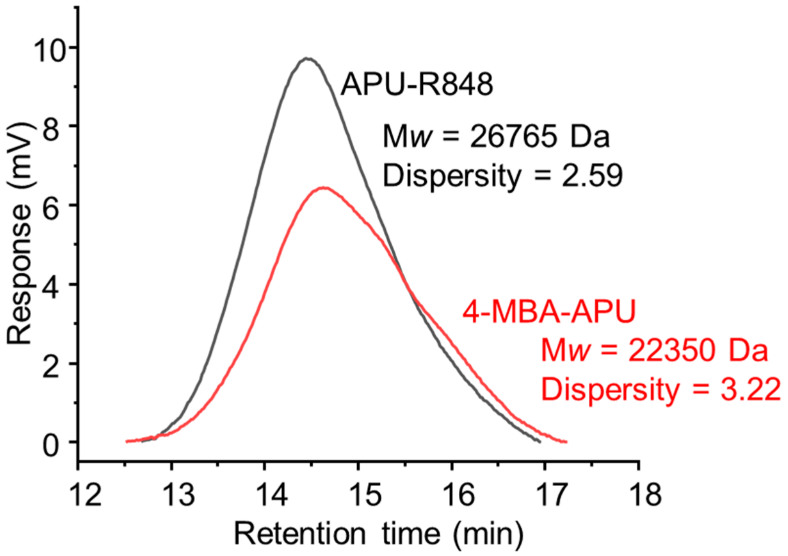

Figure S4. Molecular weight characterization of the 4-MBA-APU by gel permeation chromatography (GPC).

Optimization of APU-R848 coating onto IONP

The procedures of coating APU-R848 polymers onto IONP were optimized by tuning various structural features. As summarized in Figure S5, factors including the ratio between arginine- and R848-containing blocks (m/n molar ratio), -(CH_2)-, polymer to IONP ratio and concentrations, *etc.*, were optimized based on the efficiency of coating, stability of coated IONP, proper content of arginine and R848. The optimized polymer structures and coating procedures (Figure S5) were adopted for the preparation of APU-R848-IONP in the *in vitro* and *in vivo* studies.

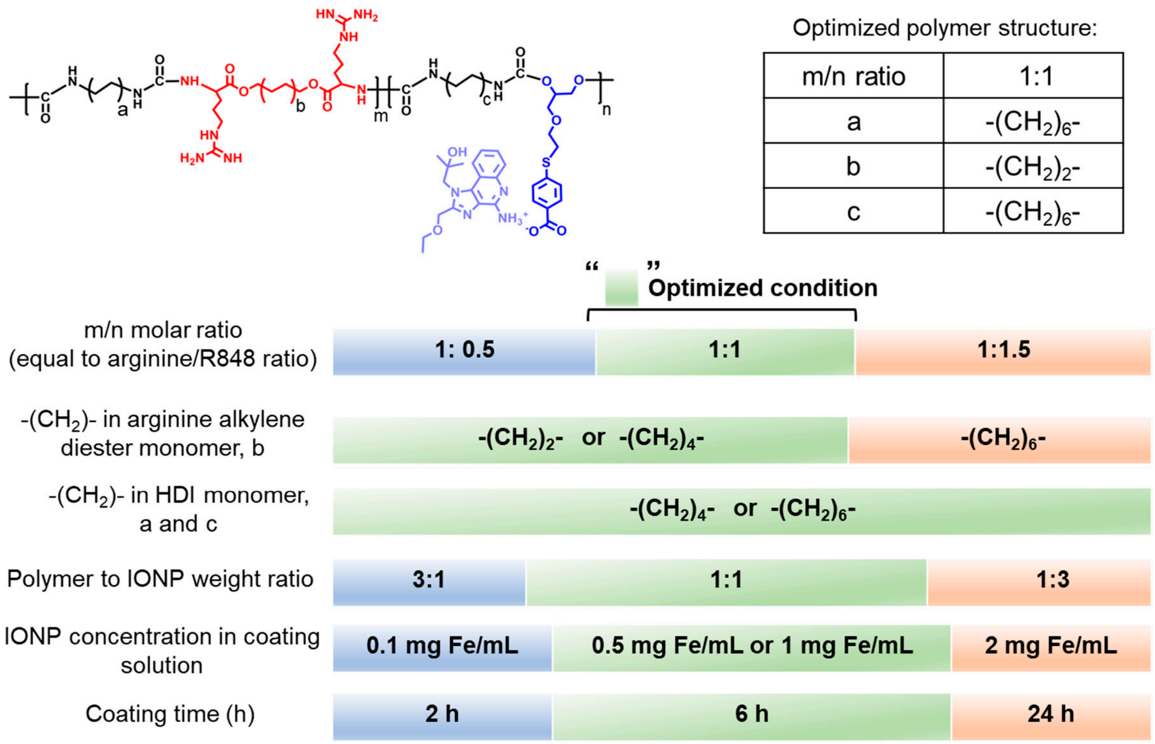

Figure S5. Tuning of APU-R848 polymer structures and coating procedures to develop optimized APU-R848 coated IONP.

Cytotoxicity study

APU-R848-IONP nanoparticles at different Fe concentrations were incubated with RAW264.7 macrophages for 48 h. The viability of macrophages was characterized via MTS assay and shown in Figure S6.

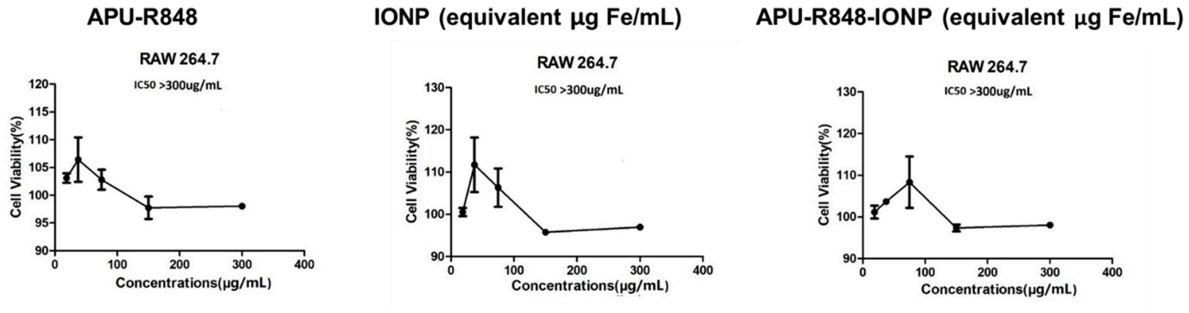

Figure S6. Cytotoxicity study of APU-R848-IONP in RAW264.7 macrophages.

Survival of 4T1-bearing mice

In addition to monitoring tumor growth, the survival of 4T1 tumor-bearing mice receiving various treatments was assessed, as shown in Figure S7 (Supporting Information). Mice in the APU-R848-IONP treatment group demonstrated an 80% survival rate by day 35. In contrast, the survival rate of mice treated with APU-R848 decreased to 50%. In the saline and IONP treated group, all mice were euthanized due to the excessive tumor burden.

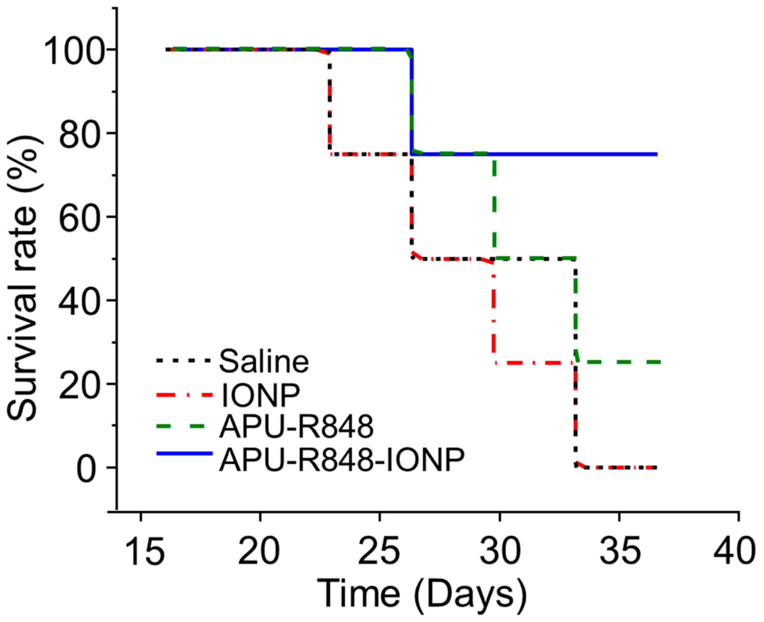

Figure S7. Survival rate of 4T1-bearing mice receiving intravenous injections of saline, APU-R848 polymer, uncoated IONP or APU-R848-IONP (n=4).

References

[1] M. He, A. Potuck, J.C. Kohn, K. Fung, C.A. Reinhart-King, C.-C. Chu, Self-assembled cationic biodegradable nanoparticles from pH-responsive amino-acid-based poly (ester urea urethane) s and their application as a drug delivery vehicle, Biomacromolecules 17(2) (2016) 523-537.

[2] M. He, C.-C. Chu, A new family of functional biodegradable arginine-based polyester urea urethanes: Synthesis, chracterization and biodegradation, Polymer 54(16) (2013) 4112-4125.
